# Simultaneous Determination of Sulfonamides Antibiotics in Environmental Water and Seafood Samples Using Ultrasonic-Assisted Dispersive Liquid-Liquid Microextraction Coupled with High Performance Liquid Chromatography

**DOI:** 10.3390/molecules27072160

**Published:** 2022-03-27

**Authors:** Yixiao Wang, Jinhua Li, Ling Ji, Lingxin Chen

**Affiliations:** 1 CAS Key Laboratory of Coastal Environmental Processes and Ecological Remediation, Shandong Key Laboratory of Coastal Environmental Processes, Research Center for Coastal Environmental Engineering and Technology of Shandong Province, Yantai Institute of Coastal Zone Research, Chinese Academy of Sciences, Yantai 264003, China; yixiaowang@yic.ac.cn (Y.W.); lxchen@yic.ac.cn (L.C.); 2 School of Source and Environment, University of Chinese Academy of Sciences, Beijing 100049, China; 3 Center for Ocean Mega-Science, Chinese Academy of Sciences, Qingdao 266071, China; 4 Yantai Oceanic Environmental Monitoring Central Station, State Oceanic Administration, Yantai 264006, China; jiling562@163.com

**Keywords:** dispersive liquid-liquid microextraction, ultrasonic-assisted, sulfonamides antibiotics, environmental water, seafood samples

## Abstract

The residues and abuse of antibiotics have seriously endangered ecological balance and human health; meanwhile, antibiotics determination is very difficult because of their low levels and multiple categories in complicated matrices. Appropriate sample pretreatment is usually imperative to enrich (ultra)trace antibiotics and eliminate matrix interference prior to chromatographic analysis. Dispersive liquid-liquid microextraction (DLLME) has become an ideal pretreatment technique owing to its simplicity, effectiveness, low-consumption, etc. In this work, an ultrasonic-assisted DLLME (UA-DLLME) was developed for the simultaneous extraction of seven sulfonamides (SAs) antibiotics in environmental water and seafood samples coupled with HPLC-DAD determination. Several parameters affecting UA-DLLME efficiency were systematically optimized, and consequently the SAs were separated and detected within 14.5 min. The obtained limits of detection (LODs) and limits of quantification (LOQs) ranged from 0.7–7.8 μg/L and 2.4–26.0 μg/L for three water samples (seawater, aquaculture wastewater and lake water) and two seafood samples (pomfrets and shrimps). High recoveries (80.0–116.0%) with low relative standard deviations (0.1–8.1%) were achieved for all the tested samples at three spiked levels. Notably, sulfadimethoxine was found at 24.49 μg/L in one seawater sample. The facile, robust and benign DLLME-HPLC method demonstrated promising perspectives for multiresidue analysis of antibiotics.

## 1. Introduction

Antibiotics are widely used in clinical treatment, agricultural and livestock production, aquaculture and so on, to control and prevent diseases and reduce economic losses [1]. Sulfonamides (SAs) are a typical group of antibiotics with the *p*-aminobenzene structure, which are prescribed for both humans and animals. Recently, the abuse of SAs has caused serious harm to ecosystem balance, such as efficacy reduction [2] and superbacteria emergence [3,4], etc. Due to the cumulative amplification effect of the food chain, SAs can be easily enriched in human and animal bodies, which poses huge potential threats to humans and other organisms [5,6]. Especially for sulfacetamide (SCT), sulfamerazine (SMR), sulfanilamide pyridine (SPD), sulfadizine (SDZ), sulfamonomethoxine (SMM), sulfamethoxazole (SMX) and sulfadimethoxine (SDM), they have a higher frequency and wider range of use and are often detected in coexistent residues, so the potential harm is greater [7,8]. To safeguard human health, a maximum residue limit (MRL) of 100 μg/kg (or 100 ppb) for the total amount of SAs in foods of animal origin has been stipulated by the European Union (EU) [9] and Ministry of Agriculture of the People’s Republic of China [10].

The residues of SAs in the environment show the characteristics of low amounts and various types. Therefore, it is urgently required to develop rapid, sensitive, affordable, and reliable methods for the concurrent determination of multiple residual SAs in complex matrices, like environmental water, food and biological samples. So far, some analytical methods have been developed for SAs determination, mainly including high-performance liquid chromatography (HPLC) [11,12], capillary electrophoresis (CE) [13], liquid chromatography-tandem mass spectrometry (LC-MS/MS) [14], and sensing analysis [15]. Often, sensing platforms are incapable for simultaneous detection of SAs. Chromatography-based techniques are more preferable for qualitative and quantitative analysis since enabling selective separation and sensitive detection of SAs residues. However, due to the low concentration of SAs in actual samples and the co-existence of matrix impurities, the direct quantitative analysis of SAs at trace levels is hardly possible to be realized. The main aims of sample pretreatment are the separation of targets from the complex matrices and the preconcentration of targets to reach a sufficient measurement level [16,17,18]. Therefore, it is of great significance to establish reliable, rapid, and efficient sample pretreatment methods for the extraction and preconcentration of (ultra)trace residues of SAs in complicated samples to reduce/eliminate matrix interference and improve detection sensitivity.

Liquid-liquid extraction (LLE) [19,20] and solid-phase extraction (SPE) [21,22,23] are two common sample pretreatment/preparation techniques that have been widely used for SAs. Amongst LLE, dispersive liquid-liquid microextraction (DLLME) has gained broad interest as an ideal pretreatment technique owing to the advantages of simplicity, low cost, effectiveness, speediness, and the small consumption of organic solvents [24,25,26,27]. Compared with SPE, DLLME is easier to implement with rapidity and more environmentally friendly, because it doesn’t need much solvent to elute targets and only needs common laboratory equipment [28]. DLLME also has a high potential for coupling with other sample pretreatment methods and analytical instruments [29,30].

So far, a number of articles have reported the preconcentration and determination of SAs by using DLLME coupling with HPLC-UV or LC-MS/MS. For example, Li et al. [31] established a simple and rapid method for the determination of thirteen SAs in water samples by utilizing ultrasonic-assisted DLLME (UA-DLLME) coupled with ultra-performance LC-MS/MS (UPLC-MS/MS). Herrera-Herrera et al. [32] developed a DLLME procedure combined with UPLC-UV to determine eleven SAs and fourteen other antibiotics in mineral and run-off waters. Ji et al. [33] synthesized deep eutectic solvent (DES) as an extractant to enrich three SAs in juice samples followed by HPLC-UV determination. However, most reported methods need more advanced instruments and/or more procedures such as synthesizing extractants. Inspired by them, it is still in high demand to develop DLLME based chromatographic methods for the convenient, rapid and sensitive determination of the multi-categories of SAs at low contents in complicated samples [34].

**Figure 1:**
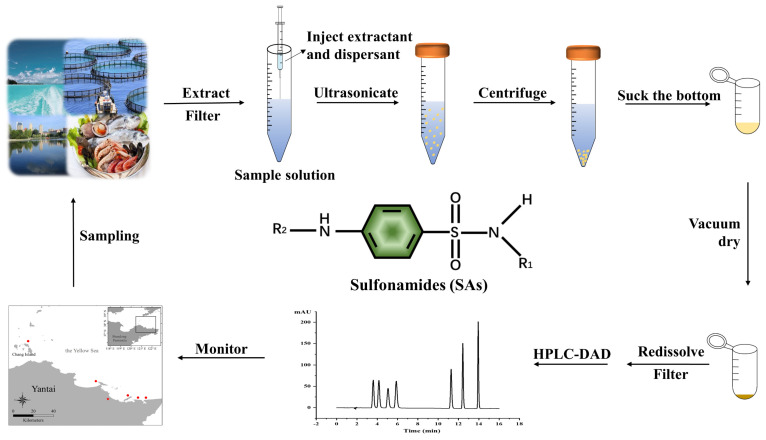
Schematic illustration of the UA-DLLME-HPLC procedure for simultaneous determination of seven SAs in environmental water and seafood samples.

Therefore, in this work, we purpose to develop a simple, fast, and efficient method based on UA-DLLME coupled with HPLC-DAD for the simultaneous effective extraction of the above-mentioned seven SAs in environmental water and seafood samples, as schematically illustrated in Figure 1. The main factors, including the kinds and volume of extractants, the kinds and volume of dispersants, sample pH, ionic strength, and the kinds of redissolved solvents were systematically optimized by using one-variable-at-a-time (OVAT). Under the optimal conditions, the analytical performance of this method, such as linear range, limits of detection (LODs), limits of quantification (LOQs) and recoveries, was evaluated in several real samples (three environmental waters and two seafoods). The proposed method was fully validated and successfully applied for the concurrent determination of seven SAs in a variety of water and food samples, presenting a good alternative to the routine monitoring of trace antibiotics in complicated matrices.

## 2. Results and Discussion

### 2.1. Optimization of UA-DLLME Conditions

To acquire the maximum efficiency of the UA-DLLME procedure, the influence of experimental parameters on the method performance was investigated. Considering that the OVAT analysis is easy and convenient, and thereby the most preferred and classical optimization choice [33,35,36,37], and because little influence occurs on the interaction among variables of the presented extraction technique, therefore the optimization process of UA-DLLME conditions was carried out by OVAT. The type and volume of extractant and dispersant, the pH of the solution, the ionic strength, and the type of redissolved solvent as significant variables were investigated, respectively. Deionized water spiked standard solution with seven SAs individual at 2000 μg/L were used for optimization experiments. The peak areas of seven SAs were used as an analytical signal to evaluate the extraction efficiency of DLLME, and all of the optimization experiments were carried out five times.

#### 2.1.1. Effect of the Type and Volume of Extractant

**Figure 2:**
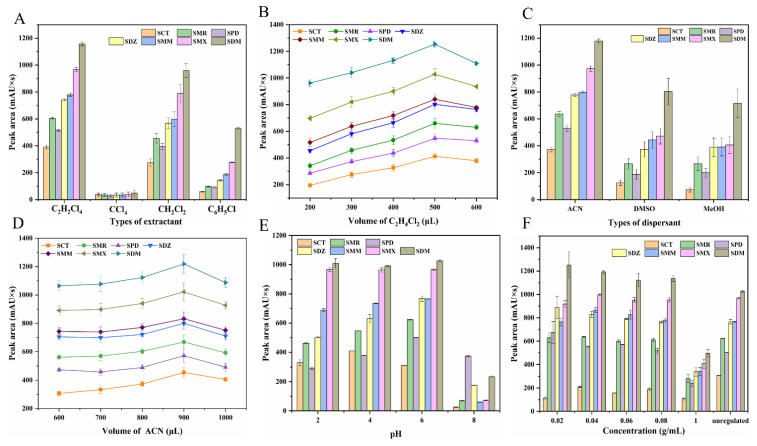
Effect of (**A**) types of extractants, (**B**) volume of C_2_H_2_Cl_4_, (**C**) types of dispersants, (**D**) volume of ACN, (**E**) pH, and (**F**) concentration of salt on the peak areas of the seven SAs. The error bar was attained from *n* = 5. Other DLLME conditions: sample solution: 5 mL; ultrasound time: 5 min; centrifuge time: 8 min; centrifuge rate: 8000 rpm.

Selecting an appropriate extractant is crucial for DLLME efficiency. The extraction solvent should be freely soluble in the dispersant, immiscible in the water phase, and have a higher affinity toward the tested analytes than the water phase [38]. Accordingly, four high-density organic solvents were investigated as extractants, including C_2_H_2_Cl_4_ (density (ρ) = 1.59 g/mL), CH_2_Cl_2_ (ρ = 1.33 g/mL), C_6_H_5_Cl (ρ = 1.11 g/mL) and CCl_4_ (ρ = 1.60 g/mL). 500 μL of the above four extractants were individually mixed with dispersant (ACN, 800 μL) and injected into a 5 mL sample solution. Figure 2A shows the peak area of the seven SAs after the DLLME procedure using the four extraction solvents. Except CCl_4_, the other three extractants had the enrichment effect on the tested SAs. As seen, the extraction efficiency of C_2_H_2_Cl_4_ was obviously higher than that of C_6_H_5_Cl and CH_2_Cl_2_. Thus, C_2_H_2_Cl_4_ was chosen as the extractant for further experiments.

The volume of extractant is also a key factor affecting extraction efficiency. The volume of C_2_H_2_Cl_4_ was tested over the range of 200–600 μL. As illustrated in Figure 2B, the highest peak area for all the seven SAs was observed when the volume of C_2_H_2_Cl_4_ was 500 μL. When the extractant’s volume was lower than 500 μL, SAs couldn’t be transferred to the organic phase effectively. But when the volume continued to increase, the extraction efficiency decreased, because a too large volume of C_2_H_2_Cl_4_ led to decreasing the concentration of SAs in the organic phase [37,38]. Thus, 500 μL of C_2_H_2_Cl_4_ was selected as the optimal volume.

#### 2.1.2. Effect of the Type and Volume of Dispersant

Suitable dispersant is essential for improving the extraction efficiency. The miscibility of dispersant in the water phase and the extractant has directly affected the formation of a cloudy solution. ACN, MeOH and DMSO were selected for the optimization process. 800 μL of the above three dispersants were individually mixed with extraction solvent (C_2_H_2_Cl_4_, 500 μL) and injected into a 5 mL sample solution. As shown in Figure 2C, when ACN was used as dispersant, the highest peak areas were obtained. ACN has good compatibility with the water phase, and a lower distribution ratio of SAs compared with that of the extractant (C_2_H_2_Cl_4_) resulted in a high extraction efficiency [37]. MeOH and DMSO possessed relatively weak dispersing capacity, and therefore, the organic phase couldn’t fully form a cloudy solution with the water phase, bringing about poor extraction efficiency. Therefore, ACN was chosen as the optimal dispersant.

The volume of dispersant was also examined ranging from 600 to 1000 μL. Figure 2D shows the peak area of the seven SAs after the DLLME procedure using different volumes of ACN. By increasing the dispersant’s volume, the extraction efficiency was gradually enhanced and finally reached a maximum at 900 μL. This may be due to that a small volume of ACN cannot fully disperse the extractant in the water phase, thus reducing the extraction efficiency [39]. Therefore, 900 μL of ACN was selected for dispersion.

#### 2.1.3. Effect of Sample Solution pH

The pH value of the sample solution is very significant for the DLLME efficiency. The pH of unregulated standard-mixture solution was 5.3 as measured by pH meter. The influence of pH within the range of 2–10 was assessed. 500 μL of C_2_H_2_Cl_4_ and 900 μL of ACN were rapidly injected into the 5 mL of standard solution with different pH. As demonstrated in Figure 2E, poor extraction efficiencies for all the seven SAs were observed at 6.0 ≤ pH ≤ 10. SAs as amphoteric compounds become acidic in water, and it is well known that when analytes are in neutral form (mostly at pH = pK_a_), they can be extracted into the organic phase more easily [40]. Consequently, by considering the pK_a_ values of the seven SAs (Figure S1, Supplementary Materials) and the pH values of real samples, the pH of the water phase in the extraction process was adjusted to 5.3.

#### 2.1.4. Effect of Ionic Strength

The ionic strength possibly plays an important role in increasing the DLLME efficiency, which is optimized commonly by adding different concentrations of salts. The addition of salt in the aqueous media could reduce the solubility of the analyte of interest in the aqueous phase, improving the extraction efficiency [41]. However, an additional excess of salt can increase the viscosity of the sample solution and suppress the analytes’ mass transfer. Moreover, the electrostatic force between the analytes and the salt ions increases, which decreases the mass-transfer ability of analytes and subsequently reducing the extraction efficiency [40,41,42,43]. Herein, the effect of ionic strength was investigated by adding NaCl (0–0.1 g/mL) into 5 mL of sample solution. As seen from Figure 2F, the ionic strength had no significant effect on the extraction efficiency of SAs, suggesting the feasibility of the developed DLLME method for analyzing samples containing salt like seawater. Therefore, no addition of salt was required during the DLLME procedure.

#### 2.1.5. Effect of Redissolved Solvent

To further ameliorate the extraction efficacy of DLLME, the type of redissolved solvents was also investigated. Based on related document literature and our experimental experiences, five kinds of redissolved solvents, including ACN, MeOH, 0.4% HAc:ACN (*v:v*, 1:1), MeOH:water (*v:v*, 1:1) and ACN:water (*v:v*, 1:1) were tested. As indicated in Figure S2A, when ACN and MeOH were used as redissolved solvents, poor separation resolution for SCT, SMR and SPD was observed. This phenomenon could be explained by the eluent strength of ACN and MeOH being much greater than that of the initial proportional HPLC mobile phase, resulting in the strong solvent effect. The strong eluting solvent participated in the elution of SAs at the beginning of the elution process, which affected the peak characteristics (shape, area, and retention time) of the first four SAs with a short retention time. Figure S2B suggests that the peak areas of all the tested SAs did not change significantly when ACN:water (*v:v*, 1:1), MeOH:water (*v:v*, 1:1) and 0.4% HAc:ACN (*v:v*, 1:1) were used as redissolve solvents, respectively. Hence, by considering the mobile phase (0.4%HAc:ACN), 300 μL of 0.4% HAc: ACN (*v:v*, 1:1) was chosen as the optimal redissolved solvent.

### 2.2. Method Performance of the UA-DLLME-HPLC-DAD

**Figure 3:**
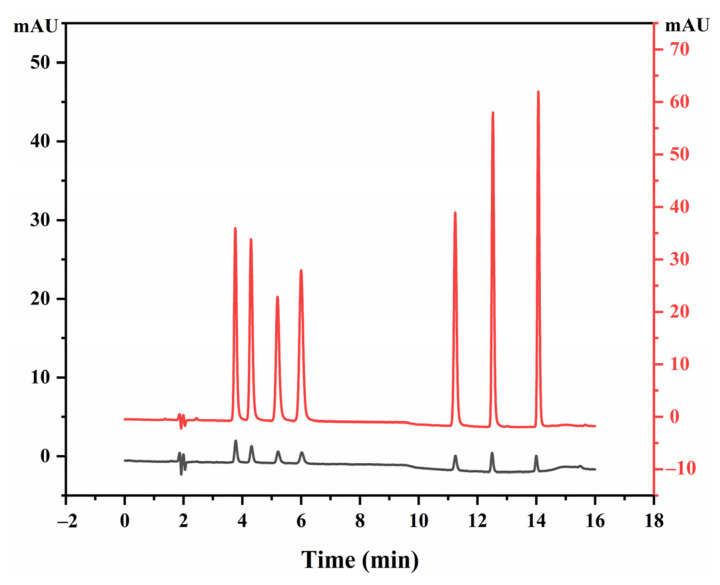
Chromatograms of the SAs standard solution individual at 500 μg/L (red) with and (black) without DLLME.

Under the optimum UA-DLLME-HPLC-DAD conditions, all the seven SAs (500 μg/L) were significantly enriched by the UA-DLLME procedure (Figure 3). The enrichment factor (EF) was calculated based on the following equation:was calculated based on the following equation:

$$EF=C_{1}/C_{0}$$

where, $C_{1}$ and $C_{0}$ are the concentrations of SAs after and before UA-DLLME, respectively. The obtained EF values for SCT, SMR, SPD, SDZ, SMM, SMX and SDM were 17.9, 18.4, 17.6, 22.4, 24.6, 27.7, and 29.0, respectively. The developed UA-DLLME-HPLC-DAD method was employed for the determination of seven SAs in environmental water samples (seawater, lake water and aquaculture wastewater) and seafood samples (pomfrets and prawns). No SAs were detected in all of the test samples. The aforementioned samples were used as blank matrices to carry out the following experiments.

Spiking blank samples (water samples and seafood samples) with concentrations of 10–5000 μg/L were used to attain matrix-matched calibration parameters. As listed in Table 1 and Table S1, the linear range of SCT in the three water samples was 10–5000 μg/L, while the linear ranges of SMR, SPD and SDZ were all within 10–5000 μg/L in seawater samples and aquaculture wastewater samples and were 5–5000 μg/L in lake water samples. The linear ranges of the other three SAs were all 5–5000 μg/L in the three water samples. Wide liner ranges were also attained for seafood samples. All linear ranges based on peak areas exhibited an outstanding correlation coefficient of *r* = 0.9999. Low LODs and LOQs were also achieved in all the actual samples based on a signal-to-noise ratio in respect to peak height (*S*/*N* = 3 and *S*/*N* = 10, respectively) according to the recommendations of the Analytical Methods Committee, UK [44]. For example, the LODs and LOQs of seven SAs were in the range of 1.3–7.8 μg/L and 4.3–26.0 μg/L in seawater samples, respectively, and in the range of 0.9–5.9 μg/L and 3.2–19.6 μg/L in pomfrets samples, respectively. The obtained LODs and LOQs of seven SAs were much lower than the MRLs in seafoods namely 0.02 mg/kg for SMR, 0.1 mg/kg for SDZ, and 0.03 mg/kg for SMM, required by Japan [45]. The reference basis on peak area and peak height, respectively, is both reasonable and feasible. The peak area refers to the total area of the peak above the background line, also known as integral strength. In quantitative calculation, integral strength (peak area) is more accurate to reflect the concentrations of analytes, because the peak width will be affected by the size of sample grain, thus affecting the peak height. On the other hand, the peak height refers to the height between the diffraction peak point and the background line, which is more sensitively attained and suitable for LOD and LOQ. Theoretically, the LODs are in the range of 10–30% of the bottom concentration of the linear range, and the LOQs are close to the bottom concentration of the linear range. Combining with the consideration of certain difference between the reference basis of peak height and peak area, several LOQs values slightly higher than the bottom concentrations are acceptable. Therefore, the results are rationally attained in Table 1 and Table S1.

The intraday and interday precision of the UA-DLLME-HPLC-DAD method was studied by using seven SAs mixed solution individual at 2000 μg/L, with five consecutive injections in one day and consecutive injections over 5fivedays, respectively. As shown in Table S2, the intraday precision (relative standard deviation (RSD), *n* = 5) values of retention time, and peak area were in the range of 0.3–1.2% and 0.3–1.5%, respectively, while the interday precision was within 0.9–2.7% and 0.8–2.4%, respectively. These results demonstrated that the developed UA-DLLME-HPLC-DAD method was qualified for accurate and reliable determination of the seven SAs concurrently in environmental water and seafood samples.

### 2.3. Evaluation of Matrix Effect

**Figure 4:**
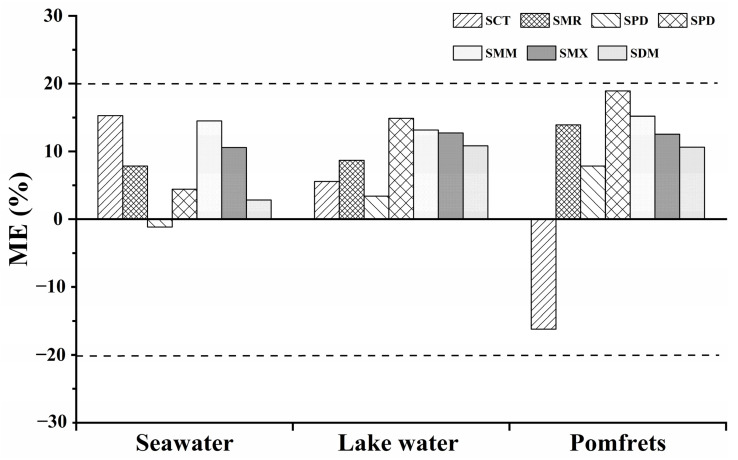
Matrix effect values for the seven SAs in seawater, lake water and pomfrets samples (*n* = 5).

As is well known, the matrix effect (ME) has an important influence on the analytical performance of a method, and sample pretreatment/preparation aims to reduce/eliminate matrix interference. In our present study, three typical samples (seawater, lake water and pomfrets) were selected to evaluate the influence of ME on the test data. As shown in Figure 4, for most SAs, the ME was positive, showing slight suppression of the analytical signal; but the analytical signal was enhanced, especially for the SCT in pomfrets, which may be related to the polarity of SCT and the pH of the extract solution [37]. As seen, the ME was obtained in the range between −20% and 20%, and therefore it can be regarded as insignificant based on the SANTE guidelines [46,47]. The low ME% indicated that the developed UA-DLLME technique effectively eliminated matrix interference and improved the detection’s accuracy, sensitivity and reliability [48,49], indicating a great application potential in actual samples.

### 2.4. Application of the UA-DLLME-HPLC-DAD Method to Seawater and Seafood Samples

To further evaluate the practical applicability of the developed method, the UA-DLLME-HPLC-UV was applied to analyze seven SAs in different types of actual samples. Figure S3 shows the chromatograms of pomfrets and seawater samples, respectively, before and after applying the UA-DLLME procedure. Obviously, all the SAs were influentially enriched after the implementation of UA-DLLME. The recovery experiments were carried out through three different concentrations of SAs (50, 500 and 5000 μg/L), which were spiked in water and seafood samples, and five parallel samples were tested. As listed in Table 2 and Table S3, satisfactory recoveries of the seven SAs were achieved for all samples at different concentrations. For example, the recoveries of seven SAs in seawater and pomfret samples were in the ranges of 80.0–106.0% and 81.1–105.2%, respectively, while RSD values ranged from 0.2–6.3% and 2.1–8.1%, respectively (Table 2). Therefore, the develop method proved applicable for the accurate quantitative determination of the seven SAs simultaneously in real samples.

**Figure 5:**
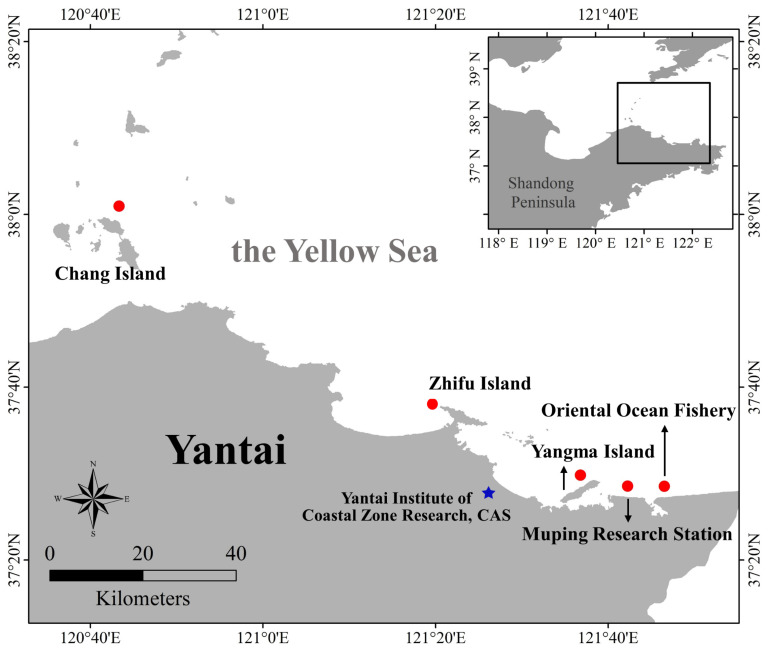
Satellite map of the five sampling locations. Seafood samples were from Zhifu Island, and water samples were from four other sampling locations, including near Chang Island, near Yangma Island, Muping Coastal Environment Research Station and Oriental Ocean Fishery.

Furthermore, more kinds of seawater and seafood samples were analyzed by using the method. The sampling locations were shown in Figure 5, including scenic spots, fishery farms and a research station. As listed in Table S4, except SDM, the other six SAs were not detected in any of the tested samples. It should be noted that the SDM was detected in seawater sampled near Chang Island, with an average concentration of 24.49 μg/L; it was identified by comparing the almost consistent retention time with that of standard solution (Figure S4) and quantified based on the regression equation of seawater in Table 1. Considering many fishery farms near Chang Island, SDM should have been widely used as a common antibiotic in aquaculture. Although the concentration is much lower than the stricter MRLs for SAs in animal products required by China and the EU [9,10], their accumulation effects cannot be neglected. Some researchers have confirmed that antibiotics can enter the food chain through ingestion by aquatic organisms with exposure experiments [50]. Moreover, some antibiotics have also been widely detected in fish (without exposure experiments) that live in aquatic environments (ocean, river) in China [51,52]. The above results indicate that SAs were probably enriched in aquatic organisms in the tested areas. As a result, the UA-DLLME-HPLC-UV possessed wide practicability and therefore can be used for routine monitoring of SAs in complicated matrices.

### 2.5. Method Performance Comparison for SAs Determination

A comparison of the developed method with other reported methods for SAs determination was presented in Table 3. As seen, lower LODs were obtained by using advanced extractants like the covalent triazine-based organic framework (CTF) [21] and the magnetic covalent organic framework (COF) [22] in SPE, magnetic ionic liquids (MILs) [11] and DES [33] in DLLME. However, the aforementioned more sensitive methods need more complex operations and synthesis processes, which would increase the workloads of the experiments [11,21,22,33]. What’s more, the performance of some synthetic extractants may be unstable due to the change of the external environment’s temperature, humidity and other objective conditions [33]. For SPE, it requires more organic reagents and an extraction device, which would be time-consuming and increase the analysis cost [21,22]. In addition, the hyphenated analytical instruments such as UPLC-MS/MS [21] and HPLC-MS/MS [22] can also improve the detection sensitivity to a large extent. But the MS detector is complex and expensive, and often unavailable in a general lab. Under the same extraction mode of UA-DLLME, our established method achieves a better enrichment effect without additional synthetic extractant [33], and detects more kinds of SAs in more kinds of real samples than that reported [33,53]. And even if compared with the two-step enrichment method [54], our method still shows comparable analytical performance. On the whole, the novelty features and advantages of our UA-DLLME-HPLC-DAD method can be summarized as follows: the method is convenient and easy to operate and popularize, and the matrix effect is effectively eliminated by the matrix-matched calibration curves established in a variety of real samples to attain reliable results; in the end, it is not only highly sensitive, accurate, stable and reliable, but also simpler, cost-saving and eco-friendly.

## 3. Materials and Methods

### 3.1. Chemicals and Reagents

Seven SAs standards, i.e., sulfacetamide (SCT), sulfamerazine (SMR), sulfanilamide pyridine (SPD), sulfadizine (SDZ), sulfamonomethoxine (SMM), sulfamethoxazole (SMX), and sulfadimethoxine (SDM), with the individual purity ≥ 98%, were all purchased from Macklin (Shanghai, China); their chemical structures and related pKa and logP values are shown in Figure S1. Chromatographic pure acetonitrile (ACN) was obtained from ANPEL Laboratory Technologies (Shanghai, China), with purity ≥ 99.9%. Chromatographic pure acetic acid (HAc) was provided by Tianjin Kemiou Chemical Reagent Co., Ltd. (Tianjin, China), with purity ≥ 98.8%. Analytical pure ACN, methyl alcohol (MeOH), dimethylsulfoxide (DMSO), tetrachloroethane (C_2_H_2_Cl_4_), chlorobenzene (C_6_H_5_Cl), dichloromethane (CH_2_Cl_2_), tetrachloromethane (CCl_4_), formic acid, *n*-hexane, and anhydrous sodium sulfate were all attained from Sinopharm Chemical Reagent (Shanghai, China), with individual purity ≥ 99.5%. Ultrapure water with the specific resistance of 18.2 MΩ.cm produced by Pall Cascada lab water purification system (Pall Corp., Westborough, MA, USA) was used for aqueous solution preparation throughout the present study. 

### 3.2. Instrument and Conditions

HPLC experiments were carried out on an Agilent high-performance liquid chromatograph (HPLC, 1260 Infinity II, Agilent Technologies, Palo Alto, CA, USA) equipped with a diode-array detector (DAD). A ZORBAX SB-C18 column (4.6 × 150 mm, 5 μm), also from Agilent Technologies, was employed. Mobile phases A and B were 0.4% HAc in ultrapure water and ACN, respectively. A gradient elution program started from 16% B, held for 6.8 min, then increased linearly to 60% B within 8 min, and held for 0.2 min before reconditioning, at a flow rate of 1.0 mL/min. The monitoring wavelength was 270 nm and the column thermostat was kept at 30 °C. The sample injection volume was 10 μL.

The Ultrasonic apparatus (KQ5200E) was bought from Kun Shan Ultrasonic Instruments Co., Ltd. (Kunshan, China). pH was adjusted by PHS-3C digital pH meter (Hangzhou Dongxing Instrument Factory, Hangzhou, China). The extracted phase was dried by a DZF-6050 vacuum drying oven (Shanghai Boxun Industry & Commerce Co., Ltd., Shanghai, China). The seafood samples (pomfrets and prawns) were crushed and homogenized with an MX-GS1 multifunctional hand-held electric cooking machine (Panasonic co., Ltd., Kadoma, Japan). The spin steaming instrument (RE-2000) was purchased from Shanghai Hongxuan Lab Instrument Co., Ltd. (Shanghai, China).

### 3.3. Preparation of Standard Solutions and Real Samples

#### 3.3.1. Standard Solutions

Standard stock solutions of seven individual SAs with the concentration of 1000 μg/mL were prepared by dissolving SAs powders in 50 mL of ACN. The standard working solutions were obtained by diluting the standard stock solutions using ultrapure water. The stock and working solutions were all stored in a refrigerator at 4 °C and used within seven days.

#### 3.3.2. Water Samples

The seawater samples were collected near the First Bathing Beach in Yantai City, which is part of the Yellow Sea. The aquaculture wastewater samples were collected from a fishery farm located in Laizhou City, Yantai City, China. The lake water samples were collected from an artificial lake located in the schoolyard of Yantai University, Yanai City, China. After the collection of water samples, they were stored in clean and dry 500 mL glass bottles. The bottles were flushed three times with corresponding water samples (lake water, seawater, and aquaculture wastewater) before sampling. All glass bottles containing water samples were kept at 4 °C and used within 24 h. Prior to analysis, all the water samples were filtered using a hydrophilic microporous filter membrane with a pore size of 0.22 μm. The above three environmental water samples were used for the method’s establishment and validation. Then for the method’s application investigation, four seawater samples were collected from different sites as follows. As shown in Figure 5, the four sampling sites were located by GPS according to the selected typical areas around Yantai City, i.e., near Yangma Island, near Chang Island, the Oriental Ocean Fishery and the Muping Coastal Environment Research Station. Sampling and sample preservation were the same as above.

#### 3.3.3. Seafood Samples

Pomfrets and prawns were bought from a local market in Laishan District, Yantai City, China for the method’s establishment and validation; two kinds of seafood samples were then collected from a large-scale seafood breeding area (near Zhifu Island) for the method’s application investigation (Figure 5). The same preparation procedures were conducted for the above two purposes. The process of extracting SAs from the shrimp and fish was based on the current National Standard of China [55], which was further optimized to be more suitable for the DLLME procedure. Different from the current national standard, an additional step aiming to remove fat in seafood samples was added. Ten pomfrets and twenty prawns were homogenized using a cooking machine, respectively. The extraction process was the same for both kinds of seafoods. Taking the pomfrets, for example, 5.0 g of the pomfrets samples was put into a 50 mL plug-centrifuge tube. Then 10 g of anhydrous sodium sulfate and 20 mL of acidified ACN (adding 1 mL to 19 mL ACN.) were added into the samples to remove water, precipitate protein, and extract SAs. The above mixture was vortexed for 1 min and ultrasonicated for 10 min followed by centrifugation for 5 min at 4000 rpm. Afterward, the supernatant was transferred into a 50 mL round-bottomed flask. Then, 20 mL acidified ACN was added to the residue for extracting again; combining with the extracting solution, the mixture was rotated to dry in a 40 °C water bath under vacuum. 1.0 mL of 20% MeOH solution was added to the mixture and the mixed solution was used to dissolve the residues by a vortex. 2.0 mL of n-hexane was added into the solution to remove fat, and then was vortexed for 30 s and centrifuged at 4000 rpm for 5 min. After centrifugation, the upper liquid was discarded, and the clear liquid was removed and dried in a vacuum drying oven at 50 °C. Finally, 900 μL of ACN was added to dissolve the residue and transfer the solution into a 10 mL tube. Before DLLME, the solution was filtered by a filter membrane with pore sizes of 0.22 μm, and then the filtered 900 μL of ACN solution was employed for the dispersion process of DLLME.

### 3.4. UA-DLLME Procedure

#### 3.4.1. Water Samples

The pH of water samples was first measured and adjusted to 5.3 by using 0.1 mol/L HCl, and then the UA-DLLME procedure was performed as follows and is schematically shown in Figure 1. A mixture containing 900 μL of ACN as dispersant and 500 μL of C_2_H_2_Cl_4_ as extractant was injected rapidly into a 10 mL tube containing a 5 mL water sample. The solution was then ultrasonicated for 5 min to form the cloudy solution. Centrifugation was then carried out at 8100 rpm for 8 min. Afterwards, the organic phase containing SAs was deposited at the bottom of the tube, and this was transferred into a 5 mL glass vial by using a micropipette. The mixture was dried at 50°C under vacuum and the residue was redissolved into 300 μL of 0.4% HAc:ACN (*v:v*, 1:1). After filtration by 0.22 μm filter membrane, the solution was injected into HPLC.

#### 3.4.2. Seafood Samples

5 mL of ultrapure water was first adjusted to pH 5.3, and then 900 μL of ACN as dispersant and 500 μL of C_2_H_2_Cl_4_ as extractant were added, respectively. The mixture was then ultrasonicated for 5 min to form the cloudy solution. After centrifugation at 8100 rpm for 8 min, the organic phase deposited at the bottom of the tube was collected and vacuum dried at 50 °C. The residue was then redissolved using 300 μL of 0.4% HAc:ACN (*v:v*, 1:1). After filtration by a 0.22 μm filter membrane, the solution was analyzed by HPLC-DAD.

### 3.5. Matrix Effect

Solvent-free matrices and extracts of real seawater, lake water and pomfrets were used to construct analytical curves. ME was evaluated by comparing the slope of analytical curves. The following equation was used to calculate the percentage *ME*% in different samples [37]:

$$ME\%=\frac{k1-k2}{k1}\times 100$$

where, *k*1 is the slope of the curve of the analyte standards prepared in ACN and *k*2 is the slope of the curve obtained by different matrices enriched by DLLME.

## 4. Conclusions

In sum, a facile UA-DLLME procedure combined with HPLC-DAD was developed for the simultaneous and sensitive determination of seven SAs in environmental water and seafood samples. The combination of C_2_H_2_Cl_4_ as an extractant and ACN as a dispersant together with ultrasonic-assistant dispersion realized the influential extraction of SAs with low LODs and LOQs. Through testing different water and seafood samples, the reliability and practicality of the method was further validated. Given the advantages, we can expect a high potential for this method in the routine monitoring of trace analytes in complicated samples. On the other hand, several limitations of the established method still exist; for example, difficulties with regard to the automating of the sample preparation procedure, the use of chloride solvents, and the further improvement of sensitivities. Accordingly, perspectives can be proposed to enhance the automation of DLLME and thereby attain the integration of sample pretreatments and instrumental analysis, to develop more eco-friendly and highly efficient solvents, and to combine DLLME with other enrichment technologies, which will elevate the extraction performance and thereby expand the applications of DLLME.

## Figures and Tables

**Table 1:** Analytical performances of the UA-DLLME-HPLC-DAD method for the determination of seven SAs in seawater and pomfret samples ^a^.

Samples	SAs	Regression Equation	*r*	Linear Range ^b^ (μg/L)	LOD ^c^ (μg/L)	LOQ ^c^ (μg/L)
Seawater ^d^	SCT	*y* = 0.1655*x* − 3.065	0.9999	10–5000	7.1	23.7
	SMR	*y* = 0.3094*x* − 8.400	0.9999	10–5000	4.9	16.2
	SPD	*y* = 0.2640*x* − 7.800	0.9999	10–5000	7.8	26.0
	SDZ	*y* = 0.3700*x* − 7.566	0.9999	10–5000	4.0	13.3
	SMM	*y* = 0.3571*x* − 2.507	0.9999	5–5000	1.9	6.5
	SMX	*y* = 0.4526*x* − 3.949	0.9999	5–5000	1.4	4.8
	SDM	*y* = 0.5507*x* − 10.243	0.9999	5–5000	1.3	4.3
Pomfret ^e^	SCT	*y* = 0.2153*x* − 5.394	0.9999	10–5000	5.0	16.6
	SMR	*y* = 0.2891*x* − 1.241	0.9999	10–5000	4.2	14.0
	SPD	*y* = 0.2405*x* − 0.614	0.9999	10–5000	5.9	19.6
	SDZ	*y* = 0.3138*x* − 7.041	0.9999	10–5000	3.3	11.0
	SMM	*y* = 0.3541*x* − 0.713	0.9999	5–5000	1.6	5.3
	SMX	*y* = 0.4428*x* − 0.734	0.9999	5–5000	1.4	4.5
	SDM	*y* = 0.5064*x* + 1.959	0.9999	5–5000	0.9	3.2

^a^ *n* = 5. ^b^ Based on peak area. ^c^ Based on peak height. ^d^ From a fishery farm in Laizhou City, Yantai City. ^e^ From a local market in Laishan District, Yantai City.

**Table 2:** Found values and recovery of the UA-DLLME-HPLC-DAD method for the determination of seven SAs in aquaculture seawater and pomfret samples ^a^.

SAs	Spiked (μg/L)	Seawater			Pomfret		
		Found (μg/L)	Recovery (%)	RSD (%)	Found (μg/L)	Recovery (%)	RSD (%)
SCT	0	ND ^b^	-	-	ND	-	-
	50	47	94.0	3.5	50	100.0	2.1
	500	510	102.0	1.9	481	96.2	2.2
	5000	4964	99.3	0.5	5002	100.0	2.5
SMR	0	ND	-	-	ND	-	-
	50	51	102.0	3.7	48	96	3.4
	500	494	98.8	1.7	511	102.2	3.1
	5000	5001	100.0	0.2	4998	100.0	3.2
SPD	0	ND	-	-	ND	-	-
	50	51	102.0	1.9	52	104.0	8.1
	500	496	99.2	1.7	493	98.6	2.8
	5000	5001	100.0	0.3	4999	100.0	2.8
SDZ	0	ND	-	-	ND	-	-
	50	42	84.0	3.0	41	81.1	4.9
	500	530	106.0	1.6	526	105.2	6.7
	5000	4998	100.0	0.3	4995	99.9	3.3
SMM	0	ND	-	-	ND	-	-
	50	41	82.0	5.6	41	82.0	3.7
	500	498	99.6	1.1	460	92.0	7.0
	5000	5000	100.0	0.3	4790	95.8	3.4
SMX	0	ND	-	-	ND	-	-
	50	40	80.0	6.3	42	84.0	6.5
	500	496	99.2	1.1	466	93.2	6.4
	5000	5000	100.0	0.4	4856	97.1	3.4
SDM	0	ND	-	-	ND	-	-
	50	44	88	1.8	41	81.7	6.8
	500	472	94.4	2.3	475	95.0	6.4
	5000	5003	100.1	2.3	4774	95.5	4.5

^a^ *n* = 5. ^b^ Not detected.

**Table 3:** Analytical performance comparison with reported methods for SAs determination.

Sample	Number of SAs	Extractant	Dispersant	Method	LOD (μg/L)	LOQ (μg/L)	*r*	Ref.
Pork, chicken and beef	14	CTF	-	SPE-UPLC-Q/TOF-MS/MS	0.05–0.54	-	>0.99	[21]
Chicken	9	MOF	-	MSPE ^a^-HPLC-MS/MS	0.8–1.6	-	>0.99	[22]
Milk	5	[C_4_MIM-TEMPO] Cl	-	In-situ MILs VA-DLLME-HPLC-DAD	0.534–0.891	1.783–2.974	>0.999	[11]
Water	11	650 μL CHCl_3_	1250 μL ACN	VA-DLLME-UPLC-DAD	0.41–8.6	1.36–28.7	>0.99	[32]
Juice	3	800 μL DES	-	UA-DLLME-HPLC-DAD	20–50	20–80	=0.9999	[33]
Water	3	650 μL CHCl_3_	1000 μL ACN	UA-DLLME-HPLC-DAD	0.07–0.25	-	>0.999	[53]
Chicken Liver	4	650 μL CH_2_Cl_2_	1000 μL ACN	DSPE ^b^-VA-DLLME-HPLC-DAD	0.4–8.4	-	>0.99	[54]
Environmental water and seafood	7	500 μL C_2_H_2_Cl_4_	900 μL ACN	UA-DLLME-HPLC-DAD	0.7–7.8	2.4–26.0	=0.9999	This work

^a^ magnetic SPE. ^b^ dispersive SPE.

## Data Availability

The data presented in this work are available in the article and supplementary materials.

## Supplementary Materials

The following supporting information can be downloaded at: https://www.mdpi.com/article/10.3390/molecules27072160/s1, Figure S1: The chemical structures and related pKa and logP values of the seven SAs; Figure S2: (**A**) Chromatograms of the seven SAs using 1 mL of MeOH (a), ACN (b) and 0.4% HAc:ACN, *v:v* = 50:50 (c) as redissolved solvents. (**B**) Effect of redissolved solvents on the peak areas of the seven SAs. Error bars are from *n* = 5. Other DLLME conditions: sample solution: 5 mL; ultrasound time: 5 min; centrifuge time: 8 min; centrifuge rate: 8000 rpm; Figure S3: Chromatograms of the seven SAs with/without DLLME in (**A**) seawater samples and (**B**) pomfrets samples. (a) blank samples with DLLME, (b) without DLLME in spiked seawater samples with seven SAs individual at 500 μg/L, and (c) after DLLME in spiked seawater samples with seven SAs individual at 500 μg/L; Figure S4: Chromatograms of (a) seawater sample near Chang Island and (b) standard solution at 100 μg/L; Table S1: Analytical performances of the UA-DLLME-HPLC-DAD method for determination of seven SAs in water and seafood samples; Table S2: Method precision of peak retention time and peak area in the standard solution; Table S3: Found values and recovery of the UA-DLLME-HPLC-UV method for the determination of seven SAs in water and seafood samples; Table S4: The results of detecting seven SAs in real samples by UA-DLLME-HPLC-DAD method.

## Sample Availability

Samples of the environmental water and seafood with limited quantity are available from the authors.
