# Supplementary material for: Simultaneous Determination of Sulfonamides Antibiotics in Environmental Water and Seafood Samples Using Ultrasonic-Assisted Dispersive Liquid-Liquid Microextraction Coupled with High Performance Liquid Chromatography
Source: Molecules. 2022 Mar 27;27(7):2160. doi: 10.3390/molecules27072160 (PMC9000397; doi:10.3390/molecules27072160)
Supplement: Supplementary file 1 [file molecules-27-02160-s001.zip › molecules-1609616-supplementary.pdf]

Supplementary Material

Simultaneous Determination of Sulfonamides Antibiotics in Environmental Water and Seafood Samples Using Ultrasonic-Assisted Dispersive Liquid-Liquid Microextraction Coupled with High Performance Liquid Chromatography

Yixiao Wang [1,2], Jinhua Li [1,2,3,*], Ling Ji [4] and Lingxin Chen [1,3]

[1] CAS Key Laboratory of Coastal Environmental Processes and Ecological Remediation,
Shandong Key Laboratory of Coastal Environmental Processes,
Research Center for Coastal Environmental Engineering and Technology of Shandong Province,
Yantai Institute of Coastal Zone Research, Chinese Academy of Sciences, Yantai 264003, China; yixiaowang@yic.ac.cn (Y.W.); lxchen@yic.ac.cn (L.C.)
[2] University of Chinese Academy of Sciences, Beijing 100049, China
[3] Center for Ocean Mega-Science, Chinese Academy of Sciences, Qingdao 266071, China
[4] Yantai Oceanic Environmental Monitoring Central Station, State Oceanic Administration,
Yantai 264006, China; jiling562@163.com
* Correspondence: jhli@yic.ac.cn

Figure S1. The chemical structures and related pK_a and logP values of the seven SAs.

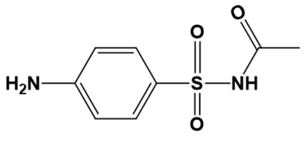

Sulfacetamide (SCT)
pK_a=5.2, logP=2.15

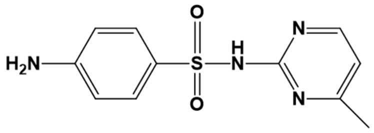

Sulfamerazine (SMR)
pK_a=6.8, logP=2.84

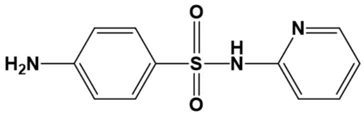

Sulfanilamide pyridine (SPD)
pK_a=8.0, logP=3.20

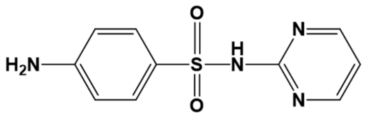

Sulfadizine (SDZ)
pK_a=6.4, logP=2.59

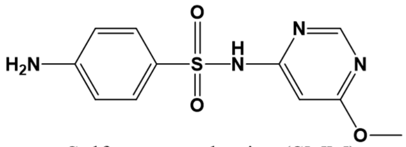

Sulfamonomethoxine (SMM)
pK_a=5.9, logP=2.60

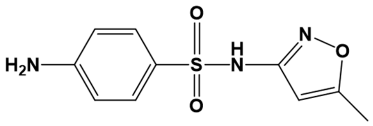

Sulfamethoxazole (SMX)
pK_a=5.6, logP=3.15

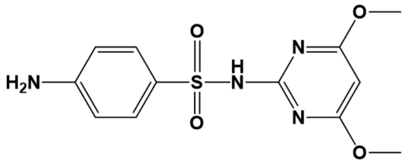

Sulfadimethoxine (SDM)
pK_a=5.6, logP=2.67

Figure S2. (A) Chromatograms of the seven SAs using 1 mL of MeOH (a), ACN (b) and 0.4% HAc:ACN, v:v=50:50 (c) as redissolved solvents. (B) Effect of redissolved solvents on the peak areas of the seven SAs. Error bars are from *n*=5. Other DLLME conditions: sample solution: 5 mL; ultrasound time: 5 min; centrifuge time: 8 min; centrifuge rate: 8000 rpm.

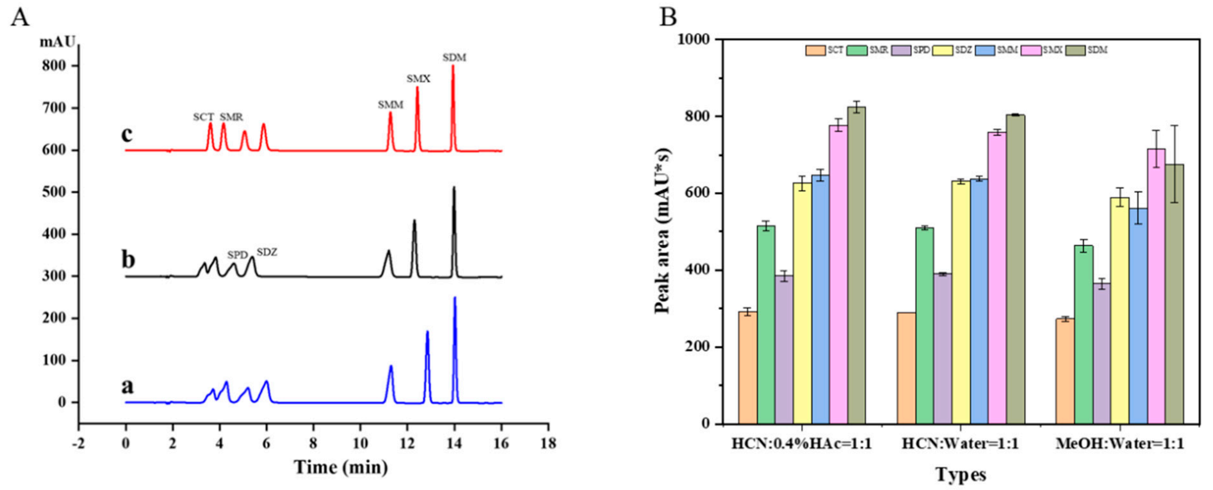

Figure S3. Chromatograms of the seven SAs with/without DLLME in (A) seawater samples and (B) pomfret samples. (a) blank samples with DLLME, (b) without DLLME in spiked seawater samples with seven SAs individual at 500 μg/L, and (c) after DLLME in spiked seawater samples with seven SAs individual at 500 μg/L.

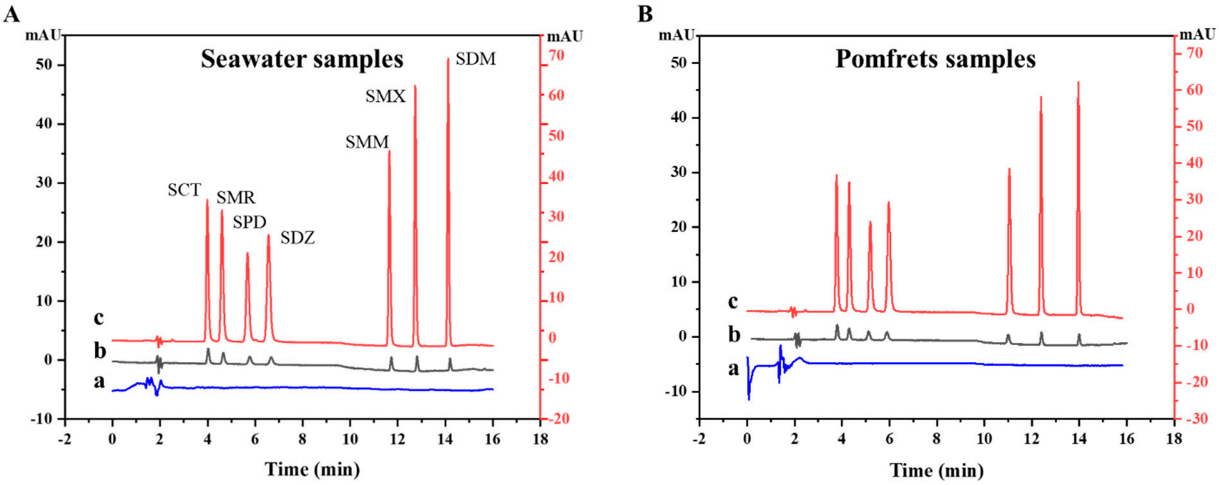

Figure S4. Chromatograms of (a) seawater sample near Chang Island and (b) standard solution at 100 μg/L.

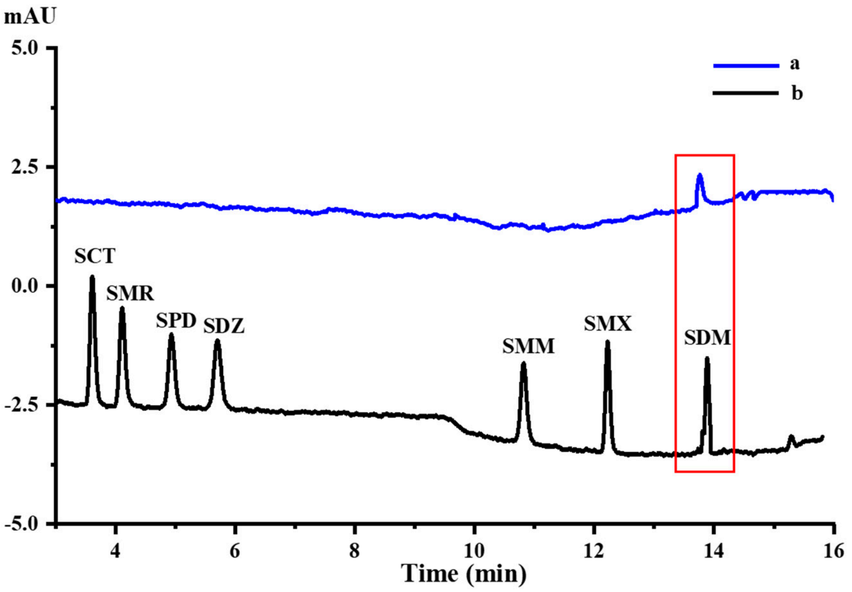

Table S1. Analytical performances of the UA-DLLME-HPLC-DAD method for the determination of seven SAs in water and seafood samples[a].

Sample	SAs	Regression equation	r	Linear range[b] (μg/L)	LOD[c] (μg/L)	LOQ[c] (μg/L)
Aquaculture wastewater[d]	SCT	$y = 0.2668x + 1.210$	0.9999	10–5000	3.5	11.6
	SMR	$y = 0.3300x + 1.226$	0.9999	10–5000	3.3	10.8
	SPD	$y = 0.2600x + 2.604$	0.9999	10–5000	5.4	18.0
	SDZ	$y = 0.3776x + 1.334$	0.9999	10–5000	4.7	15.5
	SMM	$y = 0.3855x + 0.721$	0.9999	5–5000	3.6	11.9
	SMX	$y = 0.4588x + 0.308$	0.9999	5–5000	2.6	8.6
	SDM	$y = 0.5066x – 6.045$	0.9999	5–5000	2.4	7.8
Lake water[e]	SCT	$y = 0.1831x – 0.237$	0.9999	10–5000	7.0	23.4
	SMR	$y = 0.3066x – 3.283$	0.9999	5–5000	4.6	15.2
	SPD	$y = 0.2521x – 2.185$	0.9999	5–5000	6.6	21.9
	SDZ	$y = 0.3294x + 0.938$	0.9999	5–5000	4.4	14.6
	SMM	$y = 0.3626x – 0.404$	0.9999	5–5000	2.1	7.1
	SMX	$y = 0.4419x – 0.448$	0.9999	5–5000	1.5	5.0
	SDM	$y = 0.5053x – 5.198$	0.9999	5–5000	1.1	3.6
Prawns[f]	SCT	$y = 0.3469x–10.559$	0.9999	10–5000	4.3	14.2
	SMR	$y = 0.3697x–1.730$	0.9999	10–5000	3.1	10.5
	SPD	$y = 0.3063x–1.612$	0.9999	10–5000	3.5	11.5
	SDZ	$y = 0.4036x–5.900$	0.9999	10–5000	3.1	10.3
	SMM	$y = 0.4195x–1.135$	0.9999	5–5000	2.1	6.8
	SMX	$y = 0.5128x–0.709$	0.9999	5–5000	1.2	3.9
	SDM	$y = 0.5652x+0.686$	0.9999	5–5000	0.7	2.4

[a] n=5.

[b] Based on peak area.

[c] Based on peak height.

[d] From a fishery farm in Laizhoiu City, Yantai City.

[e] From an artificial lake in Yantai University.

[f] From a local market in Laishan District, Yantai City.

Table S2. Method precision of peak retention time and peak area in the standard solution.

SAs	RSD (%, *n*=5)			
	Intraday		Interday	
	Retention time	Peak area	Retention time	Peak area
SCT	1.1	0.7	2.7	1.4
SMR	0.9	0.7	1.6	2.4
SPD	1.1	0.5	2.3	1.7
SDZ	0.8	0.6	1.5	1.2
SMM	0.5	1.5	1.3	0.8
SMX	1.2	0.3	0.9	1.1
SDM	0.3	0.7	1.1	1.6

Table S3. Found values and recovery of the UA-DLLME-HPLC-UV method for the determination of SAs in water and seafood samples[a].

SAs	Spiked (μg/L)	Aquaculture wastewater			Lake water			Prawns		
		Found (μg/L)	Recovery (%)	RSD (%)	Found (μg/L)	Recovery (%)	RSD (%)	Found (μg/L)	Recovery (%)	RSD (%)
SCT	0	ND[b]			ND			ND[a]		
	50	47	94.0	3.9	47	94.0	5.6	57	114.0	3.8
	500	509	101.8	2.2	503	100.6	3.3	481	96.2	1.5
	5000	4999	100.0	1.0	5000	100.0	0.6	5002	100.0	0.4
SMR	0	ND			ND			ND		
	50	48	96.0	1.4	55	110.0	5.6	47	94.0	6.1
	500	509	101.8	1.3	474	94.8	2.1	529	105.8	0.8
	5000	4999	100.0	0.6	5003	100.1	1.2	4998	100.0	0.8
SPD	0	ND			ND			ND		
	50	47	94.0	6.6	57	114.0	2.9	49	98.0	4.5
	500	496	99.2	2.2	480	96.0	3.5	523	104.6	3.5
	5000	5001	100.0	2.0	5002	100.0	0.7	4998	100.0	0.4
SDZ	0	ND			ND			ND		
	50	47	94.0	2.0	50	100.0	2.0	58	116.0	6.7
	500	502	100.4	2.0	502	100.4	3.7	489	97.8	0.7
	5000	5000	100.0	0.7	5000	100.0	0.8	5001	100.0	0.5
SMM	0	ND			ND			ND		
	50	48	96.0	2.3	49	98.0	2.5	50	100.0	3.0
	500	503	100.6	0.6	498	99.6	2.7	493	98.6	1.8
	5000	5000	100.0	1.8	4959	99.2	0.2	5001	100.0	0.3
SMX	0	ND			ND			ND		
	50	49	98.0	3.1	49	99.0	4.8	51	102.0	3.4
	500	499	99.8	1.2	497	99.3	3.8	493	98.6	1.4
	5000	5000	100.0	2.0	5000	100.0	0.4	5001	100.0	0.1
SDM	0	ND			ND			ND		
	50	58	116.0	6.8	53	106.9	1.4	49	98.0	4.3
	500	466	93.0	6.8	458	91.7	1.7	485	97.0	1.4
	5000	5003	100.1	0.8	5004	100.1	1.1	5002	100.0	0.2

[a] *n*=5.

[b] Not detected.

Table S4. The results of detecting seven SAs in real samples by UA-DLLME-HPLC-DAD method[a].

Samples	Sampling location	Found (μg/L)
Seawater	Near Yangma Island	ND[b]
	Near Chang Island	24.49 (SDM)
	Oriental Ocean Fishery[c]	ND
	Muping Research Station[d]	ND
Seafood	Near Zhifu Island (the Yellow Sea)	ND

[a] *n*=3.

[b] Not Detected.

[c] Oriental Ocean Trepang Breeding Base.

[d] Muping Coastal Environment Research Station, Chinese Academy of Sciences.
